# A Twisting Diagnosis: A Case Report of Double Intussusception Uncovering an Unexpected Malignant Melanoma

**DOI:** 10.7759/cureus.87398

**Published:** 2025-07-06

**Authors:** Lauren Lim, Graham R Eyeington, Taylor Nicely, Chloe Nicely, Christopher Keeler

**Affiliations:** 1 College of Medicine, Lake Erie College of Osteopathic Medicine, Bradenton, USA; 2 Department of Biology, University of Central Florida, Orlando, USA; 3 Department of General Surgery, AdventHealth Waterman, Tavares, USA

**Keywords:** bowel obstruction, double simultaneous intussusception, intussusception, malignancy, melanoma, metastasis

## Abstract

We present the case of a 63-year-old female with a history of hypothyroidism, gastroesophageal reflux disease, hemorrhoids, hyperlipidemia, iron-deficiency anemia, and previously excised melanoma, who presented with 10/10 constant, dull right upper quadrant and epigastric pain, accompanied by nausea and vomiting. The patient had switched from prescribed iron supplements to over-the-counter ferrous sulfate tablets several weeks prior to admission due to gastrointestinal discomfort. Previous upper and lower endoscopies had shown benign polyps without bleeding, and she was scheduled for a capsule endoscopy by her gastroenterologist to evaluate her anemia and ongoing GI discomfort. On examination, she appeared in mild distress, with dry mucous membranes and abdominal tenderness with guarding. Laboratory tests revealed a hemoglobin level of 6.2 g/dL (previously 10.8 g/dL) and significantly elevated liver function tests (aspartate aminotransferase (AST) 677 U/L, alanine aminotransferase (ALT) 210 U/L). A CT scan of the abdomen and pelvis with IV contrast revealed two separate long-segment intussusceptions: one in the proximal jejunum and one in the distal ileum, both without discrete lead points on imaging. Given her history of melanoma, there was concern for metastatic disease. She underwent exploratory laparotomy with bowel resection and biopsy, which confirmed metastatic melanoma. This case highlights the rarity of dual-site intussusception in adults and underscores the importance of evaluating for malignancy as an underlying cause.

## Introduction

Intussusception is the process by which a portion of the intestine telescopes into itself, typically distal to an idiopathic or pathological lead point. Peristalsis may draw the lead point forward, causing the invagination of the affected segment into another segment of the bowel [1]. When this occurs, the blood supply to the affected segment may become compromised, leading to ischemia and, eventually, necrosis. Bowel perforation and sepsis are also potential complications. It is important to note that presenting patients may remain afebrile until the onset of necrosis and intestinal perforation [2]. Intussusception may be difficult to diagnose in adults, considering that the most common presenting symptom is abdominal pain, currently the most frequent complaint seen in the emergency department. Other common complaints include crampy abdominal pain, bilious vomiting, bloating, and occasionally bloody stool [3]. If not emergently treated, intussusception can lead to poor patient outcomes due to sepsis, necrosis, and hemorrhage.

In adults, intussusception accounts for 1 to 5% of mechanical bowel obstructions. Of those cases, 77% are associated with an underlying malignancy serving as a pathological lead point [4]. Other risk factors include post-surgical adhesions, anatomical variations, endometriosis, fibroids, celiac disease, Crohn’s disease, IgA vasculitis, gastrostomy tubes, and even jejunostomy tubes [3]. Adenocarcinoma is a commonly observed culprit [4]. Primary adenocarcinoma is the most common malignant lead point in the colon, while metastatic disease, often from adenocarcinoma of another primary site, is the most common malignant lead point in the small intestine [5]. In contrast to treatment in children, adults typically undergo surgery with bowel resection [3]. Recently, there have been discussions regarding the increasing incidence of intussusception in patients with AIDS. This may be attributed to the higher rates of infectious and neoplastic conditions seen in this population. Common lead points in these patients include Kaposi sarcoma, lymphoid hyperplasia, and Non-Hodgkin lymphoma [4].

Intussusception can be classified based on location and etiology. It may have benign (e.g., polyps, Meckel’s diverticulum), malignant, or idiopathic causes. Intussusception limited to the small bowel is considered entero-enteric, while colo-colic is confined to the large bowel. It is also possible to see an ileo-colic presentation, in which the distal ileum prolapses into a segment of the ascending colon [4]. Most cases of intussusception occur in the small bowel, while large bowel involvement is less common [2]. In the case of this patient, there were two separate segments of intussusception located in the proximal jejunum and distal ileum, classifying it as two entero-enteric intussusceptions.

As mentioned above, it is highly difficult to diagnose intussusception in adults from physical examination alone due to non-specific symptoms. The exam may reveal generalized or localized abdominal pain, distension, and decreased bowel sounds. If intestinal ischemia is present, the patient may experience disproportionate pain [2]. For confirmation of the diagnosis, abdominal CT is the imaging modality of choice. On the sagittal view of a CT scan, intussusception may present as a “target” sign, representing bowel thickening from the two layers of bowel telescoping into one another. The segment may also appear as a sausage-shaped mass on coronal and sagittal views [1]. In children, abdominal ultrasound may be used, and it may also be employed in adults if a palpable abdominal mass is present [5].

For symptomatic adults with intussusception, treatment typically involves exploratory laparotomy or laparoscopy to resect lead-point masses and ischemic bowel. Unlike in children, air and contrast enemas are not commonly used for preoperative reduction in adults due to the increased risk of perforation and potential seeding of tumor cells [5]. Preoperative reduction may be considered in patients with a previously diagnosed benign lesion or those at high risk of developing short-gut syndrome following resection [5]. In adults with evidence of non-obstructing lesions on CT scan and no obvious symptoms, intervention is not imperative [5]. In patients aged 60 years and older, or in those with colonic lesions, bowel resection is strongly recommended due to the increased risk of malignancy [5]. Since the patient in this case was 63 years old, symptomatic, and had a past medical history of excised melanoma of the back, exploratory laparotomy with bowel resection and tissue biopsy was performed by the general surgeon.

## Case presentation

The patient was a 63-year-old female with a past medical history of hypothyroidism, gastroesophageal reflux disease, hyperlipidemia, melanoma of the lower back (treated and excised in 2021), and iron-deficiency anemia, who presented to the ED with the chief complaint of right upper quadrant abdominal pain. She reported experiencing this pain for about a month but decided to come to the ED due to worsening symptoms. She rated the pain as 10/10 and described it as constant and dull, associated with nausea, vomiting, and decreased appetite. She denied any weight loss. She was unaware of any family history of colon cancer. Her family history was positive for breast cancer, “skin cancer,” brain aneurysms, ischemic heart disease, and diabetes. Physical examination revealed dry mucous membranes and abdominal tenderness in the right upper quadrant and epigastric area, with guarding.

In the ED, the patient was administered a 1000 mL bolus of 0.9% sodium chloride, a 1000 mg injection of acetaminophen, a 4 mg IV injection of ondansetron, iopamidol, a 40 mg injection of pantoprazole, and 112 mcg of levothyroxine. Laboratory tests, radiologic imaging, and an EKG were also ordered. The complete blood count revealed abnormal findings, including a hemoglobin level of 6.2 g/dL (down from 10.8 g/dL a month prior). The complete metabolic panel showed transaminitis with an aspartate aminotransferase (AST) of 677 U/L, alanine aminotransferase (ALT) of 210 U/L, and alkaline phosphatase of 199 U/L. The patient’s complete blood count results are shown in Table 1.

**Table 1:** Lab values. MCV: Mean corpuscular volume; MCH: Mean corpuscular hemoglobin; MCHC: Mean corpuscular hemoglobin concentration; RDW: Red cell distribution width; MPV: Mean platelet volume; CBC: Complete blood count.

CBC parameter	Reference range	Patient value on admission
WBC	4.80-10.80 × 10³/µL	6.37 × 10³/µL
RBC	4.20-5.40 × 10⁶/µL	2.59 × 10⁶/µL
Hemoglobin	12.0-16.0 g/dL	6.2 g/dL
Hematocrit	37.0-47.0%	21.40%
MCV	80.0-99.0 fL	82.6 fL
MCH	27.0-33.0 pg	23.9 pg
MCHC	32.0-36.0 g/dL	29.0 g/dL
RDW	11.9-17.7%	14.80%
Platelet Count	148-426 × 10³/µL	220 × 10³/µL
MPV	7.4-10.4 fL	9.9 fL

**Figure 1:**
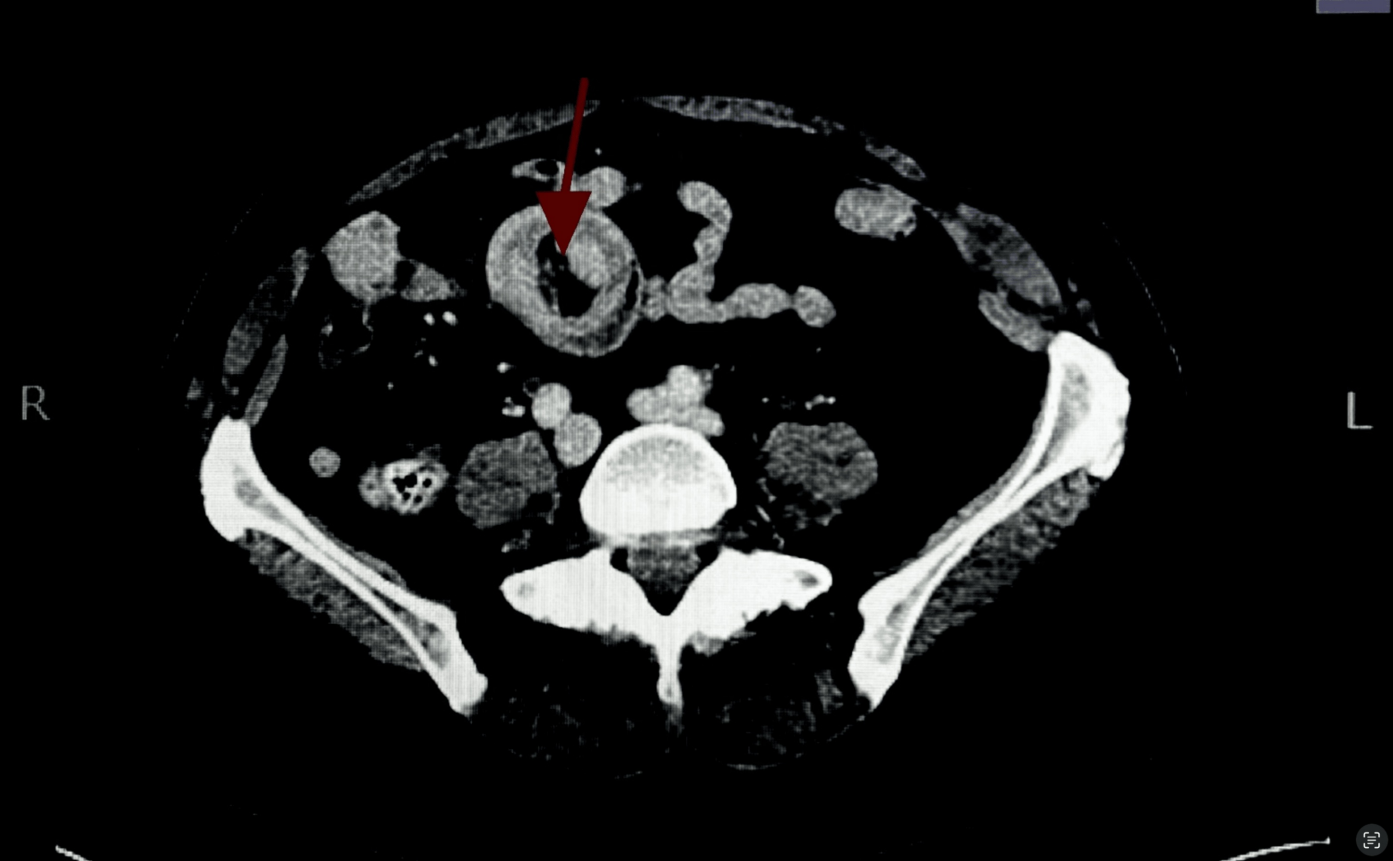
Axial view of the abdomen. The red arrow points to an intussusception of the ileum, demonstrating the characteristic “target sign.”

**Figure 2:**
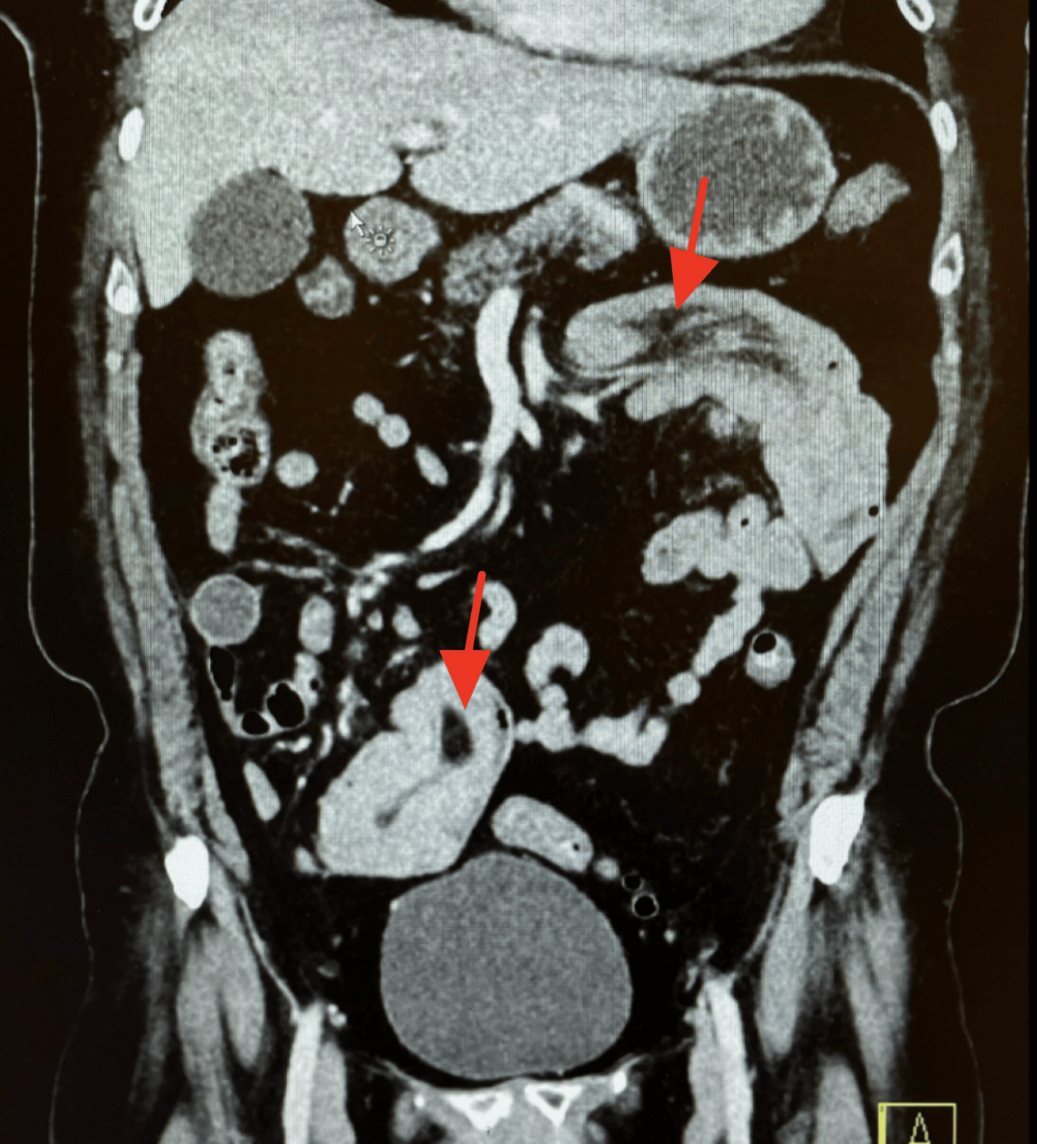
Anteroposterior (AP) view of the abdomen. The top red arrow points to a long-segment small bowel intussusception involving the proximal jejunum, which is telescoped into a more distal segment of small bowel. The total diameter of this intussusception is 4.2 cm. The bottom red arrow indicates a second segment of small bowel intussusception in the right lower quadrant, measuring up to 6.5 cm in length.

A CT scan of the abdomen and pelvis with IV contrast revealed scattered left-sided diverticula without diverticulitis. There was a long segment of small bowel intussusception within the proximal jejunum. The total diameter of the intussusception was 4.2 cm, with no discrete lead point observed. Additionally, a second segment of small bowel intussusception was identified in the right lower quadrant, measuring up to 6.5 cm in length with hyperattenuating contents or small bowel thickening seen within the segment, potentially representing a mass or collapsed bowel loops. Magnetic resonance (MR) enterography was ordered to follow up on the long-segment intussusceptions found on the prior CT. It revealed a long segment of small bowel intussusception in the left upper quadrant, measuring approximately 13.3 cm, still with no identifiable lead point or mass. In the right lower quadrant, there was a 7.1 cm segment of distal ileum intussusception with wall edema and a probable 3.0 × 4.4 cm isointense, mildly enhancing mass, potentially representative of a small bowel adenocarcinoma or neuroendocrine tumor. A magnetic resonance cholangiopancreatography (MRCP) was ordered due to the patient’s abnormal liver function tests and revealed mild intrahepatic and extrahepatic biliary ductal dilatation without evidence of choledocholithiasis or obstruction of the common bile duct. Given the patient’s past medical history of melanoma of the lower back, metastatic disease as a potential lead point could not be excluded. General surgery was consulted, and the decision was made to proceed with an exploratory laparotomy with small bowel resection and tissue biopsy to determine whether her melanoma had recurred or whether a new primary or benign process had developed. The patient was placed on a clear liquid diet, and symptom management was continued prior to surgery. Figure 1 reveals the “target sign,” a characteristic image seen on ultrasound or CT scans of intestinal intussusception. It appears as concentric, alternating echogenic and hypoechoic bands, resembling a target [5]. Figure 2 reveals both segments of intussusception on an anteroposterior (AP) view of the CT scan.

The patient underwent an exploratory laparotomy for evaluation of suspected small bowel intussusception. Upon entry into the abdomen, a thorough inspection revealed no abnormalities of the liver, spleen, stomach, or colonic gutters. The entire small bowel was then examined, starting at the ligament of Treitz.

A long-segment jejuno-jejunal intussusception was identified approximately 6-8 cm distal to the ligament of Treitz. The segment was successfully reduced, revealing a mass within the proximal jejunum serving as the lead point. The bowel was viable, with no signs of necrosis or hemorrhage. Further evaluation of the small bowel revealed an additional short-segment ileo-ileal intussusception approximately one foot proximal to the terminal ileum. The second intussusception also had a mass acting as a lead point. Two more small lesions, one just proximal and one distal to the ileal intussusception, were found, totaling four small bowel tumors. Only two were associated with intussusception.

Surgical resection was performed at both intussusception sites. A segment of jejunum containing the first mass was resected with appropriate margins, followed by a functional side-to-side, isoperistaltic jejunojejunal anastomosis using a GI anastomosis stapler and hand-sewn closure of the common channel. The second resection involved the ileal segment containing the second mass and intussusception. A traditional stapled side-to-side anastomosis was performed at this site as well, with reinforcement of the staple line and closure of the mesenteric defect.

Two Jackson-Pratt drains were placed near the anastomoses in the right and left upper quadrants. The abdomen was irrigated and closed in layers. The patient tolerated the procedure well and was transferred to the post-anesthesia care unit in stable condition.

**Figure 3:**
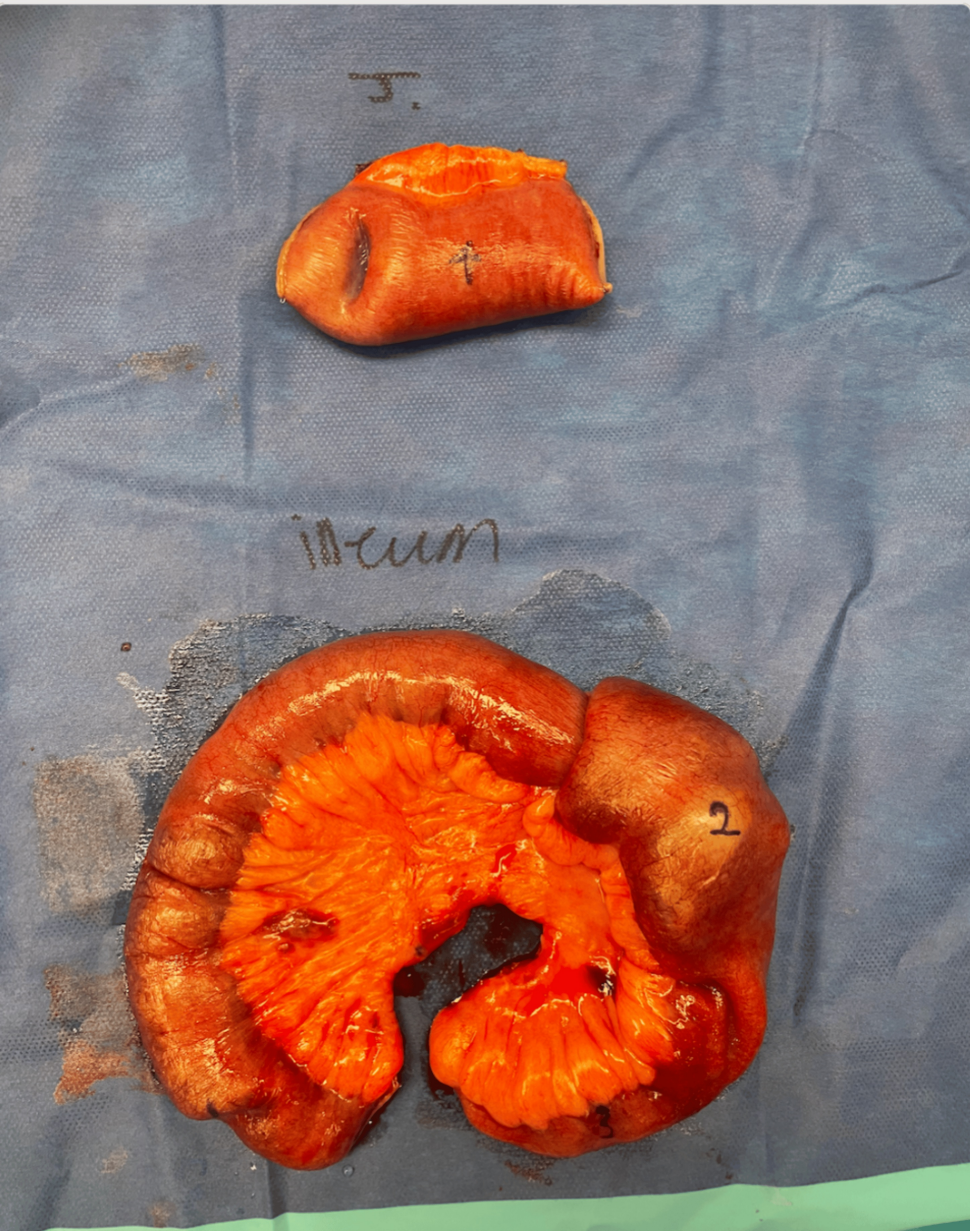
Photograph showing the four masses identified during exploratory laparotomy and small bowel resection. The top resected intussuscepted segment was from the proximal jejunum, while the bottom segment was from the distal ileum. The mass labeled number four likely acted as the lead point in the jejunum, and the mass labeled number two was most likely the lead point in the distal ileum. The masses labeled one and three in the ileum were suspected to be unrelated to the intussusception.

The resected segments containing the four masses were sent to pathology, and the diagnosis of malignant melanoma was confirmed. Unfortunately, due to the pathology report being completed in a different city and through a different hospital system, we are unable to share the specific details of the pathology report. However, the patient was referred to outpatient oncology for a follow-up appointment after the diagnosis of malignant melanoma was made. In Figure 3, we present the four masses that were found in the patient’s small bowel. The singular mass found in the proximal jejunum is labeled number four, which likely acted as the lead point. In the distal ileum, one of the masses is labeled number two, which most likely served as the lead point in that segment. The two additional masses in the distal ileum, labeled numbers one and three, most likely had no direct association with the intussusception. It is important to note that the patient’s iron-deficiency anemia may have resulted from chronic, occult GI blood loss due to mucosal ulceration or friability at the site of the metastatic melanoma causing the intussusception. Following resection, we anticipated improvement in hemoglobin and iron parameters, contingent on the absence of ongoing occult bleeding or malabsorption.

## Discussion

Adult intussusception is a rare clinical entity, representing only 1% to 5% of all cases of bowel obstruction [1]. Unlike pediatric cases, which are typically idiopathic or related to benign conditions, the majority of adult intussusceptions have an identifiable pathological lead point, most commonly malignant tumors [1,5]. In our patient, two discrete small bowel intussusceptions were found, both with confirmed malignant lead points, which revealed metastatic melanoma. This is an exceptional case of double entero-enteric intussusception with a metastatic etiology.

Melanoma is known for its ability to metastasize to the GI tract, though such presentations are uncommon and often diagnosed late in the disease course [6]. The small bowel is the most frequent site of GI metastasis in melanoma, but these lesions often remain clinically silent or present with vague symptoms such as abdominal pain, anemia, or obstruction [6]. In our case, the patient’s severe anemia, right upper quadrant pain, and markedly elevated transaminases raised concern for an underlying malignancy, despite the absence of discrete lead points on initial imaging.

This case emphasizes several important clinical considerations. First, it highlights the diagnostic challenge posed by adult intussusception, especially when imaging fails to clearly identify a mass. Although CT and MRI are the most effective imaging modalities for identifying intussusception and possible lead points, their sensitivity can be limited when tumors are isodense or small [1]. Surgical exploration remains essential in such cases, particularly in symptomatic adults, where the probability of malignancy is high. In adults, intussusception is typically caused by an underlying structural lesion such as a tumor, and the first-line treatment is surgical resection of the affected bowel segment. When a metastatic lesion, such as malignant melanoma, is identified intraoperatively as the lead point, resection without prior reduction is generally recommended to prevent intraluminal dissemination and to ensure complete removal of the tumor.

Second, this case underscores the importance of maintaining a high index of suspicion for metastatic melanoma in patients with a history of cutaneous melanoma, regardless of the time elapsed since primary tumor excision. GI metastases may appear years after initial treatment and may serve as the first sign of recurrence. Additionally, our patient had multiple metastatic deposits, with only two acting as lead points for intussusception, which aligns with literature reporting multifocal involvement of the small bowel in melanoma metastasis [6].

Our observations mirror a report of a 69‑year‑old man with prior cutaneous melanoma who developed jejunojejunal intussusception from a metastatic deposit, where nonspecific GI symptoms and imaging demonstrated obstruction requiring resection to confirm melanoma as the lead point [6]. In our case, metastatic melanoma also produced multiple small bowel lead points but uniquely caused two simultaneous entero-enteric intussusceptions, adding an extra layer of complexity to both diagnosis and surgery. These cases highlight the importance of considering metastatic melanoma in the differential diagnosis of adult intussusception, especially in patients with a history of melanoma, regardless of the time elapsed since initial diagnosis.

Our patient can be compared to another case report, highlighting the rarity of primary intestinal melanoma presenting with intussusception, though there are notable differences in patient demographics, clinical presentation, and management approaches. In the case by Kouladouros K et al., a 42-year-old female with no prior history of malignancy presented acutely with abdominal pain, nausea, and vomiting. Imaging revealed ileocolic intussusception, and emergency laparoscopy with bowel resection was performed. Histopathological analysis confirmed primary intestinal melanoma [7]. In contrast, our case involved a 77-year-old female who presented with chronic anemia and intermittent abdominal pain, with initial endoscopic evaluations failing to yield conclusive findings. Following surgical intervention and small bowel resection, histopathology revealed primary enteroenteric intussusception involving both the jejunum and ileum. Both cases demonstrate the diagnostic challenges in identifying primary intestinal melanomas, particularly when no cutaneous lesions are present. While the patient in the other case presented with acute obstructive symptoms, our patient had a more subtle, chronic presentation, highlighting the variable clinical manifestations of this rare condition. Additionally, our patient presented with double intussusception, whereas the other case involved a single segment of intussusception.

The management of both cases emphasized the importance of surgical intervention, with bowel resection being a key step in confirming the diagnosis. These cases underscore the need for clinicians to remain vigilant in considering primary intestinal melanoma in the differential diagnosis, especially given its rare presentation and the absence of obvious cutaneous signs.

Interestingly, another studied case involved a patient with recurrent intussusception. In contrast to our patient, this case described an adult with simultaneous multiple intussusceptions caused by multifocal non-Hodgkin’s lymphoma (NHL), a hematologic malignancy. While both cases involved adult patients presenting with multiple intussusceptions, the underlying etiologies differed significantly [8]. Our case highlights the rare occurrence of primary intestinal melanoma leading to simultaneous intussusception, whereas the case published in the West African Journal of Radiology underscores the role of multifocal NHL as a cause of simultaneous multiple intussusceptions. Both cases emphasize the importance of considering a broad differential diagnosis when evaluating adult patients with multiple intussusceptions, as these presentations can be indicative of underlying malignancies with distinct pathophysiologies.

Lastly, dual-site intussusception remains an exceedingly rare finding, with few reported cases. The coexistence of two separate intussusception sites with malignant lead points in different small bowel segments raises the question of whether earlier imaging or surveillance in melanoma survivors may improve detection of subclinical metastases. Although there are no current guidelines supporting routine GI surveillance in asymptomatic melanoma patients, this case contributes to the growing body of evidence that such metastases, while rare, are clinically significant.

## Conclusions

This case study emphasizes the importance of maintaining a broad differential diagnosis when evaluating nonspecific gastrointestinal symptoms in adults, particularly in patients with a remote history of malignancy. It also highlights the challenges associated with diagnosing intussusception in adults. These challenges are exemplified by this rare case of dual-site intussusception secondary to metastatic melanoma. Despite initial imaging failing to identify definitive lead points, surgical exploration revealed four intraluminal tumors, two of which served as lead points for intussusception. Clinicians should remain aware that metastatic melanoma can recur many years after initial treatment, underscoring the need for long-term follow-up and ongoing clinical vigilance. Given the multifocal nature of gastrointestinal metastases from melanoma, clinicians should also recognize the potential for delayed and nonspecific presentations, and consider early surgical consultation in similar clinical scenarios.
